# Nanostructured POSS Crosslinked Polybenzimidazole with Free Radical Scavenging Function for High-Temperature Proton Exchange Membranes

**DOI:** 10.3390/nano16030164

**Published:** 2026-01-26

**Authors:** Chao Meng, Xiaofeng Hao, Shuanjin Wang, Dongmei Han, Sheng Huang, Jin Li, Min Xiao, Yuezhong Meng

**Affiliations:** 1 The Key Laboratory of Low-Carbon Chemistry & Energy Conservation of Guangdong Province/State Key Laboratory of Optoelectronic Materials and Technologies, School of Materials Science and Engineering, Sun Yat-Sen University, Guangzhou 510275, China; mengch6@mail.sysu.edu.cn (C.M.); haoxf03@outlook.com (X.H.); wangshj@mail.sysu.edu.cn (S.W.); huangsh47@mail.sysu.edu.cn (S.H.); 2 GAC AION New Energy Automobile Co., Ltd., Guangzhou Automobile Group Co., Ltd., Guangzhou 511424, China; lijin53@gac.com.cn; 3 Institute of Chemistry, Henan Provincial Academy of Sciences, Zhengzhou 450014, China; 4 School of Chemical Engineering and Technology, Sun Yat-Sen University, Zhuhai 519000, China; handongm@mail.sysu.edu.cn

**Keywords:** proton exchange membrane fuel cell, polybenzimidazole, crosslinking, high-temperature proton exchange membrane, nanoscale polyhedral oligomeric silsesquioxane

## Abstract

High-temperature proton exchange membranes (HT-PEMs) are critical components of high-temperature fuel cells, facilitating proton transport and acting as a barrier to fuel and electrons; however, their performance is hampered by persistent issues of phosphoric acid leaching and oxidative degradation. Herein, a novel HT-PEM with abundant hydrogen bond network is constructed by incorporating nanoscale polyhedral oligomeric silsequioxane functionalized with eight pendent sulfhydryl groups (POSS-SH) into poly(4,4′-diphenylether-5,5′-bibenzimidazole) (OPBI) matrix. POSS, a cage-like nanostructured hybrid molecule, features a well-defined silica core and highly designable surface organic groups, offering unique potential for enhancing membrane performance at the molecular level. Through controlled reactions between sulfhydryl groups and allyl glycidyl ether (AGE), two functional POSS crosslinkers—octa-epoxide POSS (OE-POSS) and mixed sulfhydryl-epoxy POSS (POSS-S-E)—were synthesized. These were subsequently used to fabricate crosslinked OPBI membranes (OPBI-OE-POSS and OPBI-POSS-S-E) via epoxy–amine coupling. The OPBI-POSS-S-E membranes demonstrated exceptional oxidative stability, which is attributed to the free radical scavenging ability of the retained sulfhydryl groups on the nano-sized POSS framework. After soaking in Fenton’s reagent at 80 °C for 108 h, the OPBI-POSS-S-E-20% membrane retained 79.4% of its initial weight, significantly surpassing both the OPBI-OE-POSS-20% and pristine OPBI membranes. The PA-doped OPBI-POSS-S-E-20% membrane achieved a proton conductivity of 50.8 mS cm^−1^ at 160 °C, and the corresponding membrane electrode assembly delivered a peak power density of 724 mW cm^−2^, highlighting the key role of POSS as a nano-modifier in advancing HT-PEM performance.

## 1. Introduction

Proton exchange membrane fuel cells (PEMFCs) are categorized into two primary types based on their operational temperature ranges: traditional low-temperature PEMFCs (LT-PEMFCs) and high-temperature PEMFCs (HT-PEMFCs). LT-PEMFCs typically function at temperatures below 80 °C, whereas HT-PEMFCs operate within a range of 100 °C to 200 °C. In comparison to LT-PEMFCs, HT-PEMFCs operating at elevated temperatures offer several advantages, notably enhanced tolerance to carbon monoxide poisoning, accelerated electrode reaction kinetics, simplified water and thermal management strategies, and potential reductions in overall system costs [1,2,3].

Polybenzimidazole (PBI)-based membranes, when doped with phosphoric acid (PA), are extensively studied for HT-PEMFCs because of their inherent mechanical, thermal, and chemical stability. Proton conductivity in PA-doped PBI membranes is directly proportional to the PA doping level. However, excessive PA doping may induce plasticization, which significantly deteriorates the membrane’s mechanical integrity, leading to the leaching of free PA. Achieving an optimal balance between proton conductivity and mechanical strength in HT-PEM remains a central challenge in current research efforts. Therefore, PBI modification strategies are pivotal in the design of improved PA-doped membranes for HT-PEMFCs, aiming to address the aforementioned limitations. Researchers have devoted significant efforts to various approaches aimed at developing PBI-based HT-PEMs that exhibit high proton conductivity, mechanical robustness, and excellent thermal and chemical stability. The most extensively explored strategies at present encompass doping [4,5,6,7,8,9,10,11,12,13,14,15,16,17], blending and composite modification [18,19,20], crosslinking modification [21,22,23,24,25,26,27,28], grafting [29,30,31], main chain structure modifications [32,33,34,35,36,37,38], and microstructural regulation [39,40].

In recent years, polyhedral sesquisiloxanes (POSS) have attracted wide attention and have been widely used in various fields [41,42,43,44,45]. The exceptional thermal and oxidative stability of POSS stems from its nanoscale Si-O cage structure. Furthermore, the ability to functionalize its eight modifiable vertices with diverse organic or reactive groups facilitates uniform dispersion within organic systems, resulting in high compatibility with organic materials [46,47]. Kim et al. [48] prepared a composite membrane based on sulfonated polyether ether ketone (SPEEK) by using the sulfonated polyhedron oligosissiloxane (POSS-SA). At 80 °C and 100% RH, when POSS-SA load is 1.5 wt%, proton conductivity reaches 0.097 S cm^−1^, and the peak power density of the assembled fuel cell is 0.65 W cm^−2^. Yang et al. [49] incorporated hydrophilic and eosinophilic sulfonic-acid-containing POSS nanoparticles (SPOSS) into aryl ether type PBI with the help of sulfonated PAEK compatible agent, and the proton conductivity of the prepared PA-doped membrane reached 0.126 S cm^−1^ at 200 °C anhydrous state. He et al. [50] synthesized octa-amino polyhedral oligomeric silsesquioxane (OA-POSS), a hydrophilic and acidophilic compound, and employed it as both a filler and crosslinker in the preparation of a polyaryl ether sulfone (PAES)-based HT-PEM. The POSS cage structure enhanced the membrane’s acid retention capabilities, while the inherent hydrophilicity of POSS facilitated the formation of a microphase-separated morphology within the membrane. Consequently, the proton conductivity was improved and reached 97.4 mS cm^−1^ at 180 °C.

The operation of fuel cells generates free radicals, including •OH and •OOH, which can initiate polymer degradation upon attacking the membrane, thereby compromising fuel cell performance and longevity [51,52]. It has been reported that sulfhydryl (-SH) compounds can trap and consume free radicals, and the oxidation resistance of membranes containing sulfhydryl compounds to Fenton’s reagents has been improved [53,54]. He et al. [55] prepared PEM with excellent oxidation stability by grafting 2-sulfhydryl-1-methylimidazole (MIm), 3-sulfhydryl-1,2, 4-triazole (MTz), and 2-sulfhydryl benzimidazole (BIm) onto the skeleton of polyaryl ether sulfone (PAES), respectively. The results demonstrated that the grafted sulfur-containing moieties functioned as free radical scavengers, markedly enhancing the oxidative stability of the PAES-R membrane and effectively mitigating its degradation. After 60 h and 100 h Fenton testing at 68 °C, the acid-doped PAES-MIm membrane maintained peak power densities of 320 mW cm^−2^ and 291 mW cm^−2^ at 140 °C, respectively.

In this work, to further enhance the oxidative stability and phosphoric acid retention of the HT-PEM, POSS-SH was synthesized. POSS-SH possesses eight vertices, each functionalized with a thiol (-SH) group, readily reactive with vinyl groups under ultraviolet irradiation. Consequently, allyl glycidyl ether (AGE) was incorporated to synthesize POSS-S-E. Upon incorporation of POSS-S-E into OPBI, the epoxide groups of POSS-S-E facilitate crosslinking with PBI via N-H reactions, while the pendant thiol groups on POSS-S-E scavenge free radicals by forming disulfide (S-S) bonds. This dual functionality enhances the oxidative stability of the composite membrane. Furthermore, the Si-O cage structure inherent to POSS contributes to enhanced phosphoric acid retention. Consequently, the phosphoric-acid-doped, POSS-crosslinked OPBI membrane is anticipated to exhibit superior mechanical properties, oxidative stability, and proton conductivity.

## 2. Materials and Methods

### 2.1. Materials

3,3″-Diaminobenzidine (DAB, 98%) was purchased from Sigma-Aldrich (St. Louis, MO, USA). (3-Sulfhydrylpropyl) trimethoxysilane(KH-590), hydrochloric acid (HCl), acetonitrile (C_2_H_3_N), diphenyl ketone (DPK), 4,4″-Oxybisbenzoic acid (OBBA, 98%), phosphorus pentoxide (98%), phosphoric acid (85% solution in water), and sodium bicarbonate (99.8%) were purchased from Aladdin (Tokyo, Japan). Tetrahydrofuran (THF), allyl glycidyl ether (AGE), methanesulfonic acid (99.5%), and N-Methyl pyrrolidone (NMP, 98%) were purchased from Macklin (Shanghai, China). All of the reagents were used as received without further purification.

### 2.2. Synthesis of OPBI

Figure S1 shows the synthesis process of OPBI, which adopts DAB and OBBA direct polycondensation method to synthesize OPBI polymer [56]. A total of 100.0 g methanesulfonic acid and 10.0 g phosphorus pentoxide were added to a 250 mL three-neck flask and stirred under nitrogen at 80 °C for 1 h until a clear solution was formed. Then DAB (2.57 g, 12 mmol) and OBBA (3.10 g, 12 mmol) were added and stirred for 3 h to form a homogeneous solution. The mixture is then heated to 140 °C and mechanically stirred for 3 h until it becomes a high-viscosity solution. The hot solution is then slowly poured into deionized water to form a brown filamentous precipitate, which is then crushed into a powder and washed to neutral with dilute sodium bicarbonate solution. The powder is then washed several times with distilled water and ethanol. Finally, the polymer was dried in a vacuum at 120 °C for 24 h.

### 2.3. Preparation of POSS-SH, POSS-E, and OE-POSS

#### 2.3.1. Preparation of POSS-SH

POSS-SH was prepared by hydrolysis condensation reaction [57]. In a 500 mL flask fitted with a magnetic stirrer, 360 mL methanol, 30 mL concentrated hydrochloric acid, and 15 mL (3-sulfhydrylpropyl) trimethoxysilane (KH-590) were mixed in a nitrogen atmosphere. The mixed solution was then reflowed for 24 h at 90 °C to ensure that the hydrolytic condensation reaction took place completely, resulting in a white viscous solid at the bottom of the solution. The crude product was washed 3 times with cold methanol to remove the unreacted KH-590, and the viscous solid was dissolved in a small amount of THF and recrystallized in acetonitrile at −18 °C. After standing overnight, white granular solid was obtained, filtered, and vacuum dried at 80 °C; white crystal POSS-SH was obtained and the yield was 22%.

#### 2.3.2. Preparation of POSS-S-E

POSS-SH and AGE were dissolved in THF and prepared into 10 wt% solution, 0.895 g of AGE solution was added into 2 g of POSS-SH solution, and 1~3 wt% of benzophenone of the total mass of the reactants was added as the photocatalyst. This was stirred at room temperature for 10 min to dissolve all benzophenone and mix the reactants evenly. At room temperature, the sulfhydrylgenesis reaction between the reactants was induced by simultaneous agitation under ultraviolet (UV) light. The reaction stopped after 1 h. The THF was dried using a gyrosteamer. The product POSS with sulfhydryl group and epoxy group was obtained, namely POSS-S-E.

#### 2.3.3. Preparation of OE-POSS

POSS-SH and AGE were dissolved in THF and prepared into a solution of 10 wt%, and 1.79 g of AGE solution was added to 2 g of POSS-SH solution, and then 1~3 wt% of benzophenone of the total mass of the reactants was added as the photocatalyst. This was stirred at room temperature for 10 min to dissolve all benzophenone and mix the reactants evenly. At room temperature, the alkene-sulfhydryl addition reaction occurred between the reactants by stirring under ultraviolet light at the same time. The reaction stopped after 1 h. The THF was dried using a gyrosteamer. The product Octa-epoxy group POSS (OE-POSS) was obtained.

### 2.4. Membrane Preparation

#### 2.4.1. Preparation of the OPBI Membrane

At 120 °C, the OPBI was dissolved in NMP to obtain 2.5% PBI solution. The membrane was prepared by solution pouring method. The PBI solution was poured on a glass plate and heated at 80 °C for 10 h and 150 °C for 3 h to make the solvent volatilize. Finally, the solvent in the membrane was completely removed with deionized water and dried at 100 °C for 24 h.

#### 2.4.2. Preparation of the OPBI-OE-POSS-x% and OPBI-POSS-S-E-x% Membrane

OE-POSS or POSS-S-E was dissolved in NMP to obtain a solution of 10 wt% OE-POSS or POSS-S-E. The OE-POSS or POSS-S-E solution was dropped into the OPBI solution (2.5 wt% NMP solution) and stirred at the same time and continued to stir for 1 h after the drip was added to make the crosslinking agent and the OPBI solution evenly mixed. The mixed solution was poured on the glass plate, and then the glass plate was placed on the heating pad. After heating at 80 °C for 12 h, the solvent was heated at 150 °C for 5 h to volatilize, and the process of in situ crosslinking was completed. Finally, the solvent in the membrane was completely removed with deionized water and then dried at 100 °C for 24 h. The resulting membranes were represented by OPBI-OE-POSS-X% or OPBI-POSS-S-E-x%, where x% represented the percentage of the crosslinking agent (OE-POSS or POSS-S-E) in the total mass of OPBI and crosslinking agent at the time of feeding. The membrane thickness ranges from 20 μm to 30 μm.

### 2.5. Characterization

#### 2.5.1. Thermal Analysis

Thermogravimetric curves were recorded from room temperature up to 800 °C at a heating rate of 10 °C/min in nitrogen atmosphere with a flow rate of 100 mL/min by a Pyris1 TGA (Perkin Elmer, Waltham, MA, USA). The samples were pretreated by heating to 100 °C at a rate of 10 °C/minute and kept for 30 min before cooling to room temperature.

#### 2.5.2. Mechanical Properties

Mechanical properties of the membranes were measured with a universal testing machine (CG10, Labthink International, Inc., Jinan, China). The membranes were cut into long rectangular shaped samples whose initial dimensions were 40 mm in length and 5 mm in width. Measurements were carried out at room temperature (RT) with a constant drawing speed of 5 mm min^−1^.

#### 2.5.3. PA Doping Treatment

Dried membranes were cut into 5 × 4 cm^2^ size specimens and the weights of individual specimens were separately noted. For phosphoric acid (PA) doping, the specimens were immersed into 85 wt% PA at 120 °C for 12 h. The membranes were taken out from the PA solution, and excess PA was removed from the membrane surface by carefully wiping the specimens with dust-free paper. After drying at 100 °C for 12 h, PA uptake and the size of each sample were measured immediately.

The PA uptake ratio of the membrane (acid doping content (ADC)) was calculated by the following equation:

$$\mathrm{Acid}\;\mathrm{doping}\;\mathrm{content}\;(\mathrm{ADC})\;(\%)\;=\;\frac{W_{2}-W_{1}}{W_{1}}\times 100$$

where $W_{1}$ is the initial weight of dried membrane and $W_{2}$ is the weight of PA-doped membrane.

The volume swelling ratio and area swelling ratio are defined as the percentage of the membrane volume augment and membrane area augment, respectively, after PA doping treatment and are calculated by the following equation:

$$\mathrm{Volume}\;\mathrm{Swelling}\;(\%)\;=\;\frac{V_{2}-V_{1}}{V_{1}}\times 100$$

$$\mathrm{Area}\;\mathrm{Swelling}\;(\%)=\frac{S_{2}-S_{1}}{S_{1}}\times 100$$

where $V_{2}$ and $S_{2}$ are the volume and area of the PA-doped membrane, respectively. $V_{1}$ and $S_{1}$ are the volume and area of the undoped dry membrane.

PA uptake and PA doping level in the unit volume (*V_doping_*) was obtained by the following equation:

$$V_{doping}(g\;{cm}^{-3})=\frac{W_{2}-W_{1}}{V_{1}}$$

#### 2.5.4. Structural Characterization

FT-IR spectra of KH-590, AGE, OE-POSS, POSS-S-E, POSS-SH, OPBI membrane, PBI- POSS-S-E-x% membrane, and PBI-OE-POSS-x% membrane were recorded on a (Perkin Elmer precisely Spectrum 100) FT-IR Spectrometer (PerkinElmer, Waltham, MA, USA).

#### 2.5.5. Oxidative Stability

To test oxidative stability, the undoped membrane samples were immersed into Fenton’s reagent (3% H_2_O_2_ solution containing 4 ppm Fe^2+^) at 80 °C for 36 h, 72 h, and 108 h; then, the samples were removed from the solution and dried in a vacuum oven at 120 °C for 12 h. Each type of membrane was tested for 3 samples, and the average value was reported. The residual weight of the samples represented the oxidative stability.

#### 2.5.6. Microscopic Morphology Characterization

TESCAN VEGA3 Scanning Electron Microscope (SEM) (TESCAN, Brno, Czech Republic) was used to observe and analyze the surface and cross-section morphology of dry membrane samples. Corresponding element analyses were conducted using an energy-dispersive X-ray (EDX) analyzer attached to the SEM. The specimens were coated with a thin layer of gold before the test.

#### 2.5.7. Gel Fraction Test

The effect of crosslinking was determined by gel fraction test. The membrane samples were immersed in NMP at 80 °C for 12 h and then removed from the solvent and dried. The gel fraction was calculated by comparing the weight of the dried sample before and after solvent extraction.

#### 2.5.8. Proton Conductivity Measurements

Proton conductivity was measured via a standard four-probe AC impedance method using a PGSTAT204 N electrochemical workstation (AUT86925, AUTOLAB, Utrecht, The Netherlands). All samples were cut to 1 cm × 4 cm size, and the average thickness of the samples was obtained by a thickness tester. The PA-doped membranes were stuck in a four-probe clamp and placed in a temperature-controlled chamber. The impedance measurement was performed from 80 to 160 °C under anhydrous conditions in a frequency range of 100 mHz to 100 kHz. The conductivity was calculated by the following formula:

$$\mathrm{Proton}\;\mathrm{conductivity}\;\sigma \;=\;\frac{L}{R\;\times W\;\times T}$$

where L is the distance between reference electrodes, R is the membrane resistance, and W and T are the width and thickness of the samples, respectively.

#### 2.5.9. Phosphoric Acid Retention

The membranes were cut into squares (2 × 3 cm^2^) and placed in an environmental chamber at 80 °C and 40% humidity and the weight of membranes was measured at different times.

#### 2.5.10. Fuel Cell Tests

The fuel cell property was assessed using single-cell stacks.

Hydrogen and oxygen without pre-humidification were applied to the fuel cell at flow rates of 80 mL min^−1^ and 160 mL min^−1^ and without back pressure. The single cell was operated at 160 °C. The area of each of the membrane electrode assemblies (MEAs) was 2.5 × 2 cm^2^. The electrodes, 1.0 mg cm^−2^ Pt/C for the anode and the cathode, were purchased from Shanghai Hensen Company (Shanghai, China). The membranes and electrodes were bonded by hot-pressing at 130 °C for 2 min with a loading of 1 MPa, and the active area of the membrane–electrode assembly (MEA) was 5 cm^2^. Polarization curves were obtained using current-step potentiometry.

## 3. Results and Discussion

### 3.1. Structure and Morphology Characterization of OPBI, POSS-SH, POSS-S-E, and OE-POSS

**Scheme 1:**
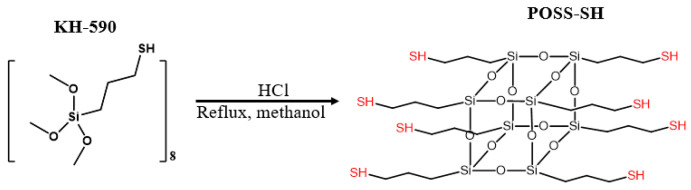
Preparation method of POSS-SH.

**Scheme 2:**
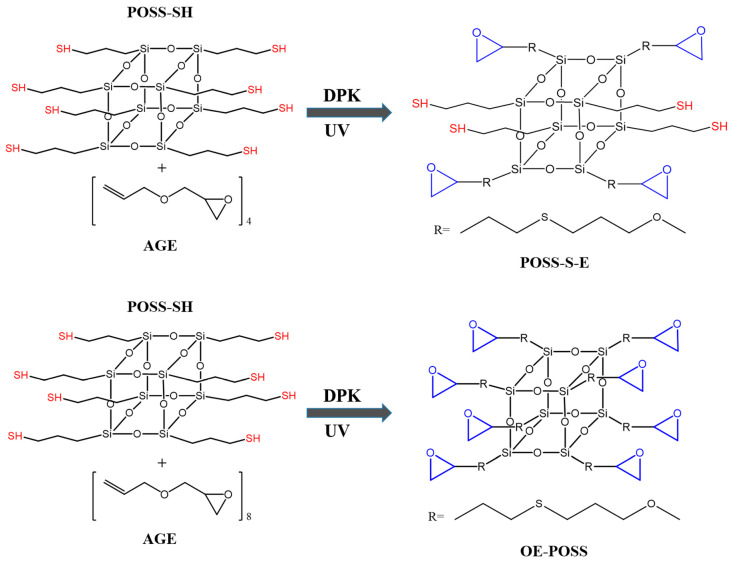
Preparation method of POSS-S-E and OE-POSS.

The ^1^H-NMR spectrum of OPBI, depicted in Figure S2, exhibits peaks consistent with the proposed molecular structure, aligning with previously published data [56]. Scheme 1 illustrates the synthesis of POSS-SH via hydrolytic condensation of thiopropyl trimethoxysilane (KH-590), yielding a white particulate solid. As shown in Scheme 2, POSS-S-E and OE-POSS were synthesized from allyl glycidyl ether (AGE) and POSS-SH via thiol-ene click chemistry. OE-POSS possesses eight epoxy groups at its vertices. POSS-S-E incorporates both sulfhydryl and epoxide moieties in a 1:1 ratio, with the epoxide acting as the crosslinking site and the sulfhydryl group serving as the free radical scavenger. Figure S3 displays the ^1^H-NMR spectra of the POSS derivatives, with the sulfhydryl proton resonance observed at 1.38 ppm. Following the thiol-ene click reaction between AGE and POSS, ^1^H-NMR spectra of POSS-S-E exhibited a diminished resonance. Conversely, the absence of a signal at 1.38 ppm in the OE-POSS spectrum indicates complete consumption of POSS-SH sulfhydryl groups and subsequent epoxy functionalization.

**Figure 1:**
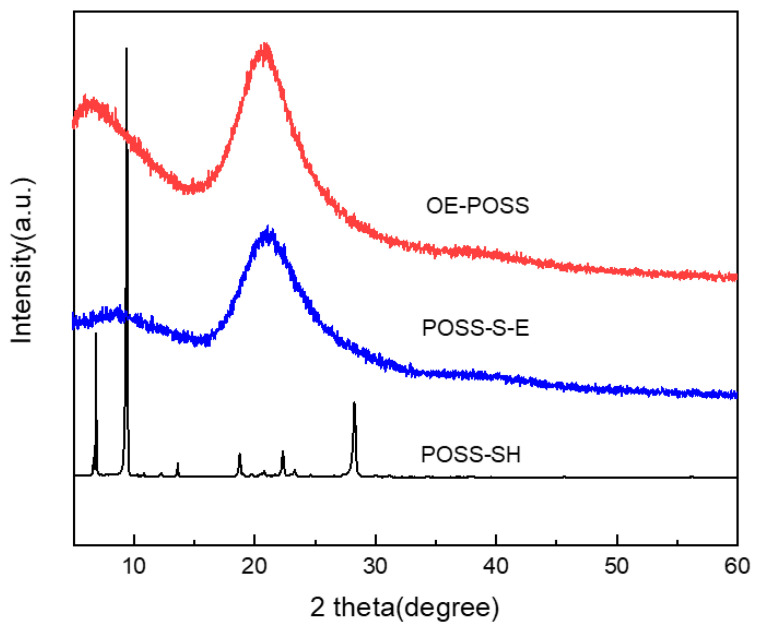
XRD patterns of POSS-SH, POSS-S-E, and OE-POSS.

**Figure 2:**
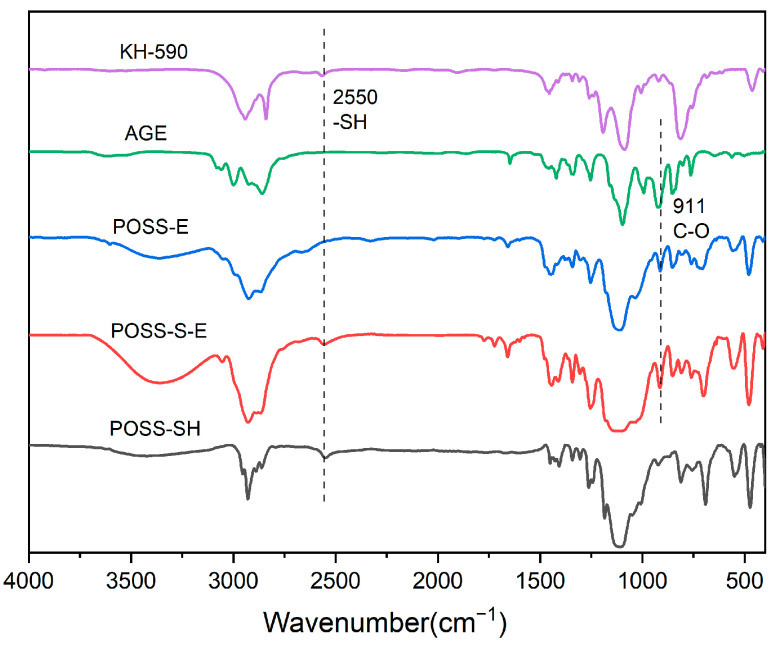
FT-IR spectra of raw materials and different POSS.

Figure 1 presents the X-ray diffraction (XRD) patterns of the three POSS compounds. The XRD spectrum of POSS-SH exhibits prominent diffraction peaks, indicative of a crystalline structure, which aligns with the literature [57]. In contrast, POSS-S-E and OE-POSS display only broad peaks in the 2θ range of 20–25°, potentially attributable to the aliphatic chains disrupting molecular ordering, leading to diminished crystallinity. Figure 2 illustrates the infrared (IR) spectra of the starting materials and POSS derivatives. KH-590, POSS-SH, and POSS-S-E exhibit -SH stretching vibration peaks at 2550 cm^−1^, confirming the presence of -SH moieties in POSS-SH and POSS-S-E. Furthermore, AGE, POSS-S-E, and OE-POSS display C-O stretching vibration peaks characteristic of epoxy groups at 911 cm^−1^, indicating successful epoxy functionalization of POSS-S-E and OE-POSS. In conclusion, POSS-SH, POSS-S-E, and OE-POSS were successfully synthesized.

**Figure 3:**
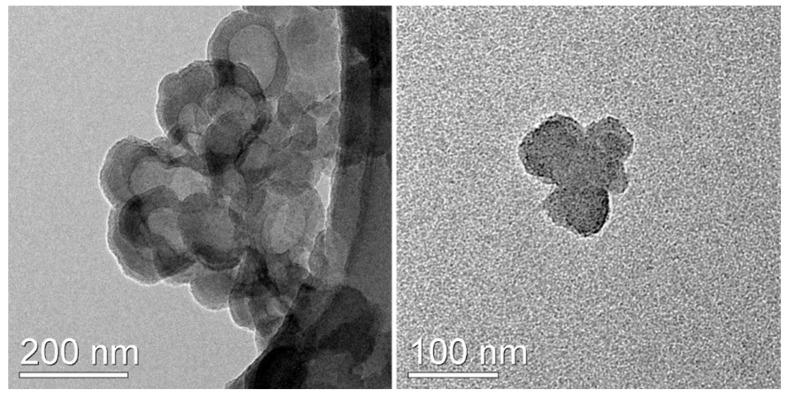
TEM image of POSS-SH.

The TEM image of POSS-SH is shown in Figure 3 The sample preparation process is to disperse POSS-SH in anhydrous ethanol, drop 10 μL of the dispersion onto the microgrid copper net, and then dry it under an infrared lamp. It can be seen that POSS-SH is composed of nanoparticles with a diameter of 30 nm to 100 nm, and these nanoparticles are agglomerated to a certain extent, forming large particles of several hundred nanometres.

### 3.2. Preparation of POSS-Crosslinked OPBI Membrane

**Figure 4:**
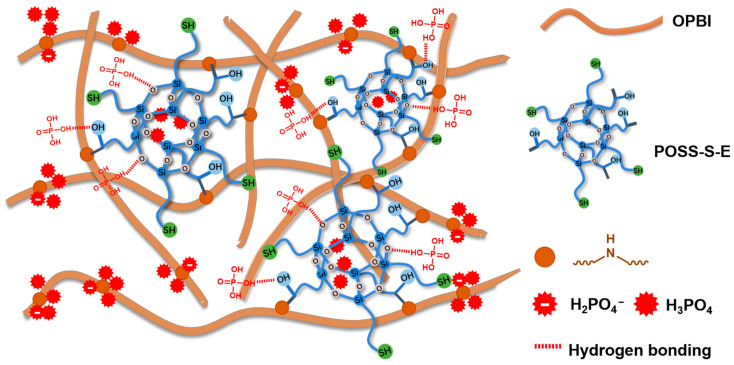
Schematic diagram of OPBI-POSS-S-E membrane structure.

**Scheme 3:**
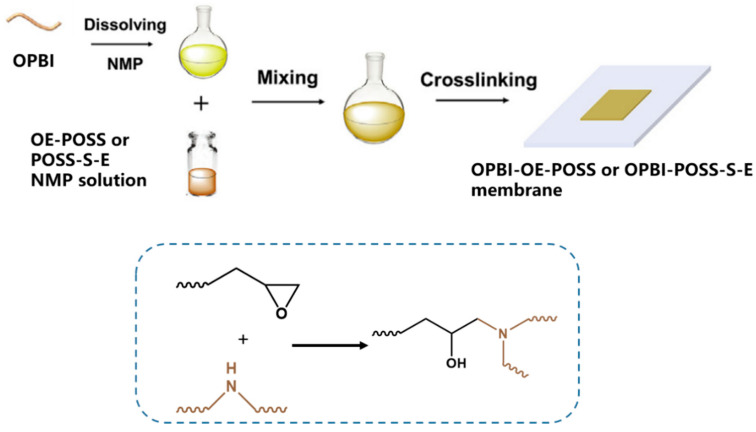
Preparation process of OPBI-OE-POSS and OPBI- POSS-S-E membranes.

As depicted in Scheme 3, POSS-crosslinked OPBI membranes were fabricated by dissolving OPBI and a crosslinking agent (OE-POSS or POSS-S-E) in NMP solvent, casting the solution onto a glass plate, and heating the plate on a hotplate. The membranes were initially heated at 80 °C for 12 h, followed by a subsequent heating step at 150 °C for 5 h. During heating, the epoxide groups on POSS react with the -NH groups on the benzimidazole ring of OPBI and undergo ring-opening reaction to complete in situ crosslinking membrane formation. As illustrated in Figure 4, the sulfhydryl groups on POSS-S-E react with free radicals forming sulfur–sulfur bonds, thus consuming free radicals. Furthermore, a robust hydrogen bond network forms between POSS and PA, decreasing free acid content and enhancing the PA retention capability of membrane.

**Figure 5:**
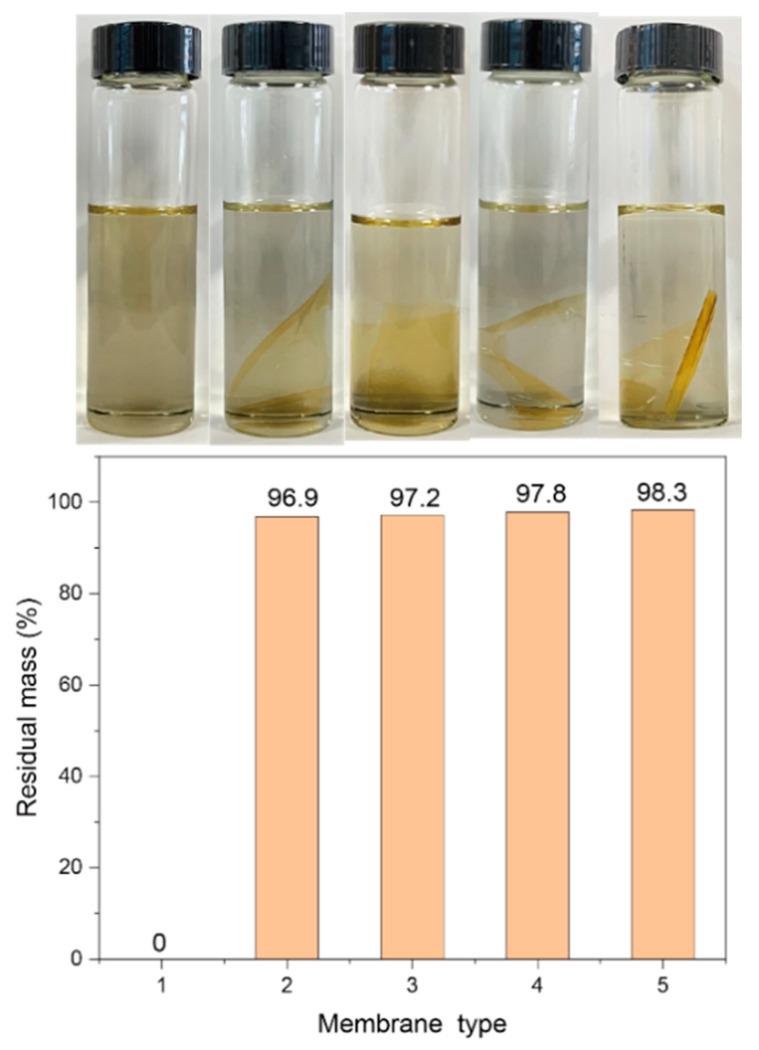
Gel fraction test of different membranes (1. OPBI; 2. OPBI-OE-POSS-10%; 3. OPBI-OE-POSS-20%; 4. OPBI-POSS-S-E-10%; 5. OPBI-POSS-S-E-20%).

Gel content test is an effective method to verify the success of membrane crosslinking. Figure 5 shows the gel content test results of different membranes. After soaking in NMP at 80 °C for 12 h, the OPBI membrane has been completely dissolved in the solvent, while other crosslinked membranes still maintain complete morphology, and each crosslinked membrane has more than 96% gel content. With the increase in POSS addition, the gel content did not change significantly, indicating that the addition of 10% POSS had fully crosslinked OPBI.

### 3.3. Structure Characterization

**Figure 6:**
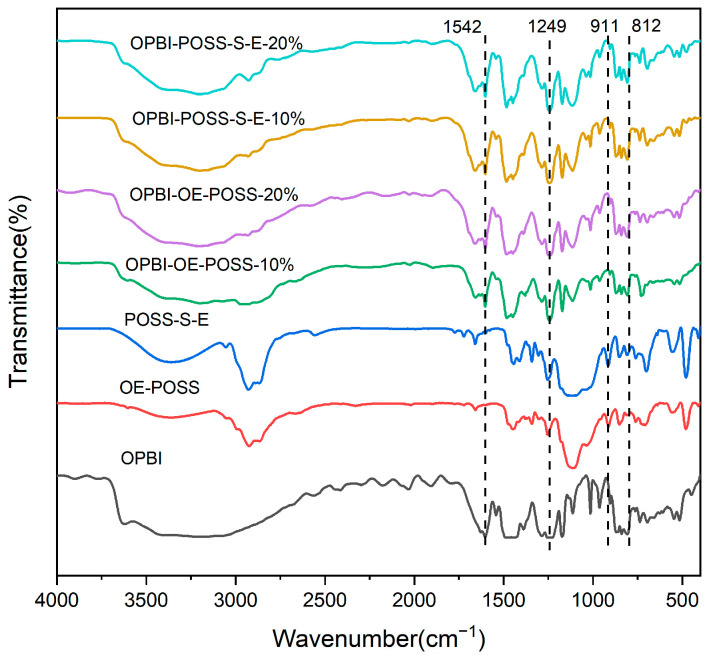
FT-IR spectra of different membranes.

The FT-IR spectra (Figure 6) revealed the presence of a C-O bond stretching vibration peak at 911 cm^−1^, characteristic of the epoxy group in OE-POSS and POSS-S-E. This peak was absent in the spectrum of the crosslinked membrane, indicating a ring-opening reaction between the epoxy group and the NH moiety of the PBI imidazole ring, confirming successful crosslinking. The gel content test also showed this. Near 812 cm^−1^ is the characteristic peak of the five-membered ring in OPBI, 1249 cm^−1^ is the stretching vibration peak of C-N, and 1542 cm^−1^ is the stretching vibration peak of –C=N– [56].

**Figure 7:**
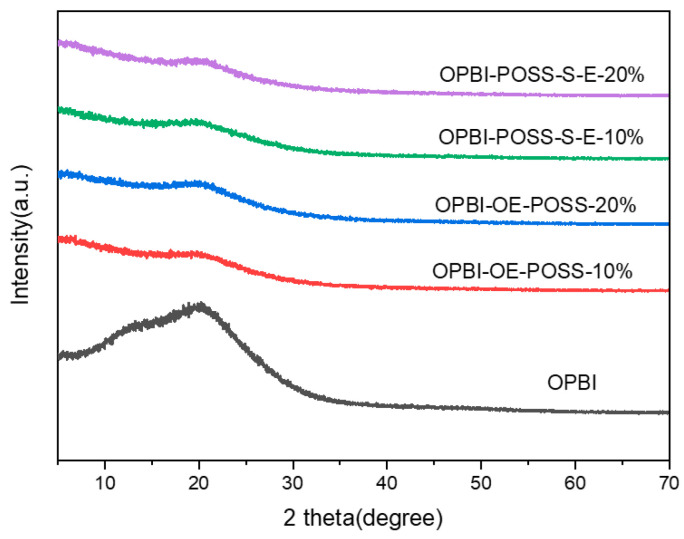
XRD patterns of different membranes.

The XRD patterns of OPBI, PBI-OE-POSS-10%, OPBI-OE-POSS-20%, OPBI-POSS-S-E-10%, and PBI-POSS-S-E-20% membranes is shown in Figure 7. The wide diffraction peak at 2θ value of about 20° is attributed to the π-π stacking of the aromatic rings, indicating that the OPBI membrane was generally amorphous and also indicating that the benzimidazole ring is parallel to the membrane surface orientation [58,59]. Compared with OPBI membranes, the peak strength of OPBI-OE-POSS and OPBI-POSS-S-E crosslinked membranes with OE-POSS or POSS-E decreased significantly, exhibiting amorphous envelope morphology. This reduction in crystallinity arises from the introduction of OE-POSS or POSS-S-E, which crosslinks the OPBI, hindering the ordered arrangement of the polymer backbone and leading to a less defined structure in the crosslinked membrane.

**Figure 8:**
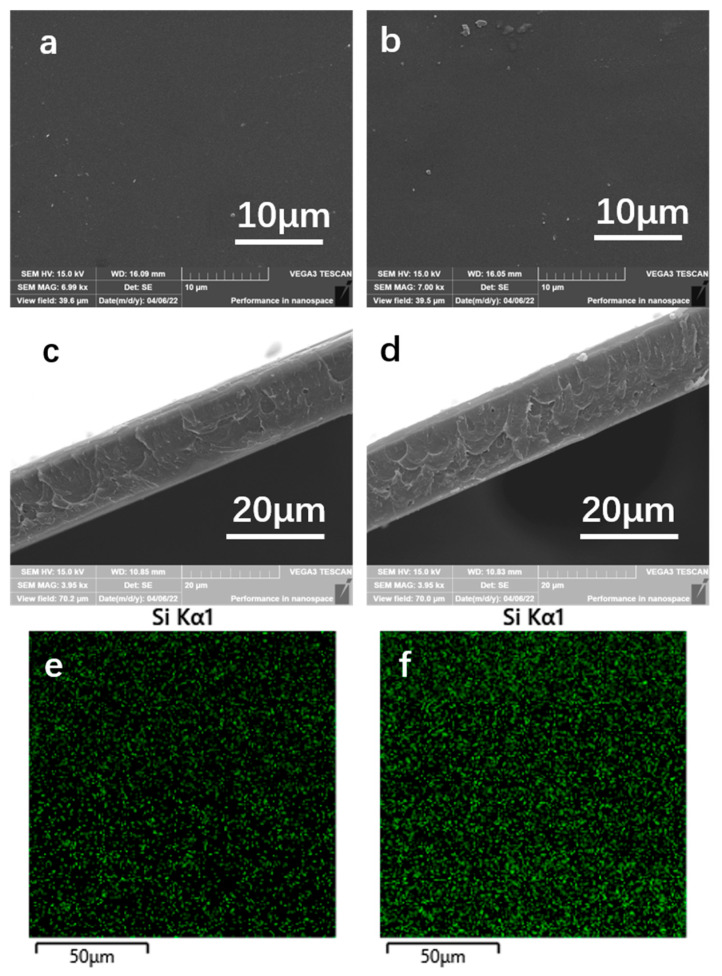
Surface and cross-section morphology images and Si mapping of OPBI-POSS-S-E-10% (**a**,**c**,**e**) and OPBI-POSS-S-E-20% (**b**,**d**,**f**) membranes.

Scanning electron microscopy (SEM) was used to characterize the surface morphology of the polymer membrane. Figure 8a,b showed the surface morphology images of OPBI-POSS-S-E-10% and OPBI-POSS-S-E-20% membranes with a magnification of 7000 times. It could be seen that the surface of the membrane was uniform and dense, with no holes or cracks. Figure 8c,d show the cross-section topography images with a magnification of 4000 times. It can be seen that the thickness of the membrane is about 20 μm, and the inside of the membrane is uniformly dense without holes. Such a membrane has good air tightness, which enables PEM to well separate the anode and cathode in the fuel cell and enables the fuel cell to have a high open circuit voltage to ensure its stable operation. Figure 8e,f show the energy spectrum plane scanning of Si elements with uniform distribution, indicating that POSS-S-E is uniformly distributed in the membrane.

### 3.4. Thermal Stability

**Figure 9:**
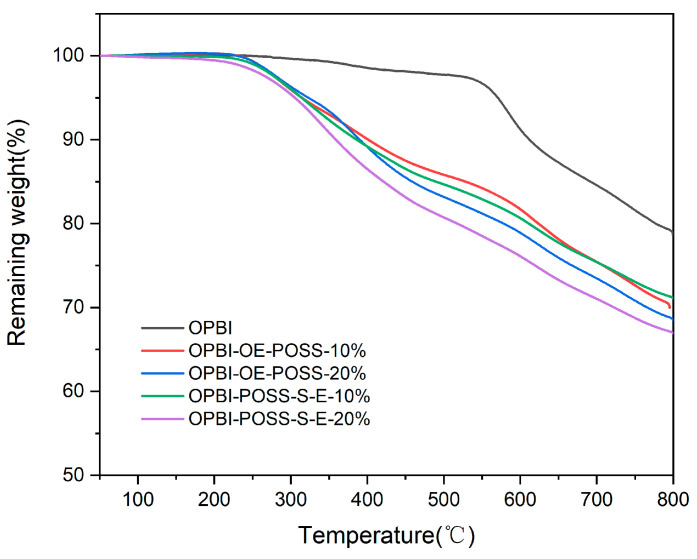
TGA curves of OPBI and crosslinked membranes.

The operating temperature of HT-PEMFC is usually between 100 and 200 °C, so the thermal stability of PEM has high requirements. Figure 9 shows the TGA curves of OPBI membrane and crosslinked membrane. OPBI membrane began to show obvious thermal weightlessness after 500 °C, which was caused by degradation of polymer main chain. The weight loss of the crosslinked membrane after 230 °C is due to the fracture and decomposition of organic groups on POSS, and the weight loss after 350 °C is due to the decomposition of siloxane. Si-O has a strong bond energy (460 kJ mol^−1^), which makes the Si-O-Si inorganic skeleton have excellent thermal stability. The T_5%_ of OPBI-OE-POSS-10%, OPBI-OE-POSS-20%, OPBI-POSS-S-E-10%, and OPBI-POSS-S-E-20% membranes are 314 °C, 323 °C, 314 °C, and 304 °C, respectively. The excellent thermal stability enables it to meet the requirements of HT-PEMFC applications.

### 3.5. Phosphoric Acid Doping and Swelling Rate

Table 1 presents the phosphoric acid (PA) doping levels and swelling ratios of the membranes, which were immersed in PA at 120 °C for 12 h. Compared to the OPBI membrane, the crosslinked membranes exhibit lower PA doping levels and swelling ratios, suggesting improved dimensional stability. The epoxy group in OE-POSS is twice that of POSS-S-E, and the theoretical crosslinking degree of OPBI-OE-POS-20% membrane should be greater than that of OPBI-POSS-S-E-20% membrane, but the phosphoric acid doping amount of the latter is smaller, indicating that its actual crosslinking degree is higher. This discrepancy may arise from the fact that not all eight epoxy groups on OE-POSS are able to react with the N-H groups on OPBI. Furthermore, the high epoxy group content in the OPBI-OE-POSS-20% membrane may promote self-polymerization reactions between epoxy groups during membrane formation [60], leading to increased agglomeration of POSS nanoparticles. Additionally, some epoxy groups may react with N-H sites on the same OPBI chain, resulting in intramolecular reactions rather than intermolecular crosslinks.

### 3.6. Phosphoric Acid Retention

**Figure 10:**
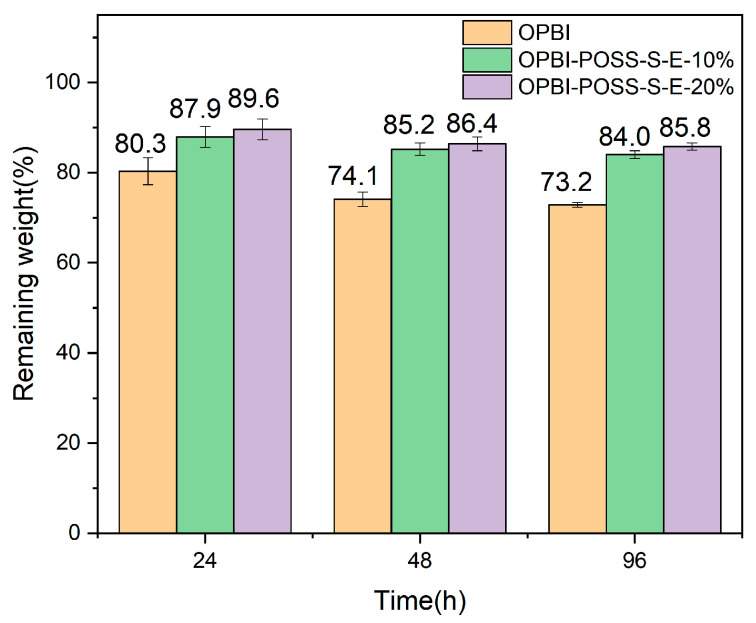
Phosphoric acid retention rate of different membranes at 80 °C and 40% RH.

It is particularly important to improve the phosphoric acid retention capacity of PA-doped PEM, which is one of the most critical factors affecting the service life of fuel cells. Figure 10 shows the phosphoric acid retention rates of different membranes tested at 80 °C and 40%RH. After 96 h, the remaining mass of PA-doped OPBI membranes is only 73.2% of that before the experiment. The residual mass of OPBI-OE-POSS-20% and OPBI-POSS-S-E-20% membranes can reach 84.0% and 85.8%, respectively, and the phosphoric acid retention rate of the two crosslinked membranes is higher than that of OPBI membranes, which is due to the crosslinked structure and the cage structure of POSS being conducive to acid retention so that phosphoric acid is not easily lost.

### 3.7. Oxidation Stability

**Figure 11:**
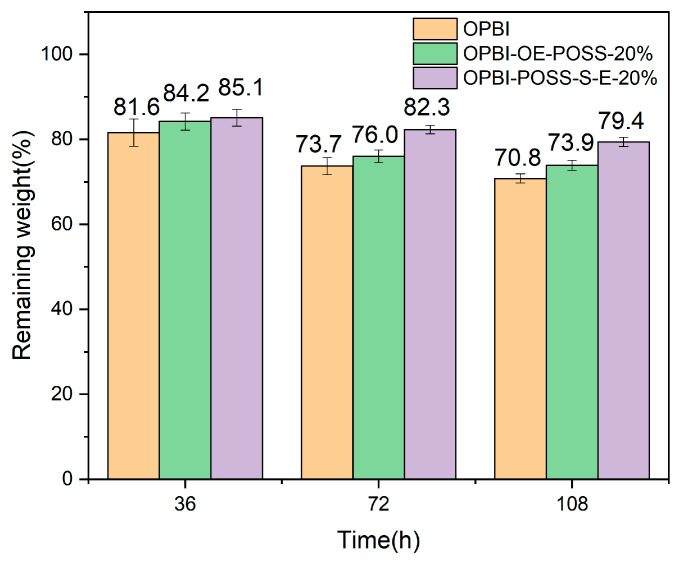
Oxidation stability of different membranes at 80 °C with Fenton’s reagent.

The oxidation stability test is a commonly employed and effective method for evaluating the long-term operational stability of HT-PEMs. Figure 11 illustrates the oxidation stability of the membranes, assessed by immersion in Fenton’s reagent at 80 °C. The crosslinked membranes exhibited a higher residual mass compared to the OPBI membrane, suggesting that crosslinking enhances oxidation stability of membrane. The residual mass of OPBI-POSS-S-E-20% membrane is greater than that of OPBI-OE-POSS-20% membrane, and the advantage is not obvious at 24 h, and the residual mass is significantly larger after 72 h, indicating that its oxidation stability is better. This is because the sulfhydryl groups present in OPBI-POSS-S-E-20% are capable of capturing free radicals from the Fenton’s reagent, thereby forming S· radicals. Subsequently, two S· radicals combine to form disulfide bonds, which plays the role of free radical elimination and improves the oxidative stability of the membrane [54,55].

### 3.8. Mechanical Properties

**Figure 12:**
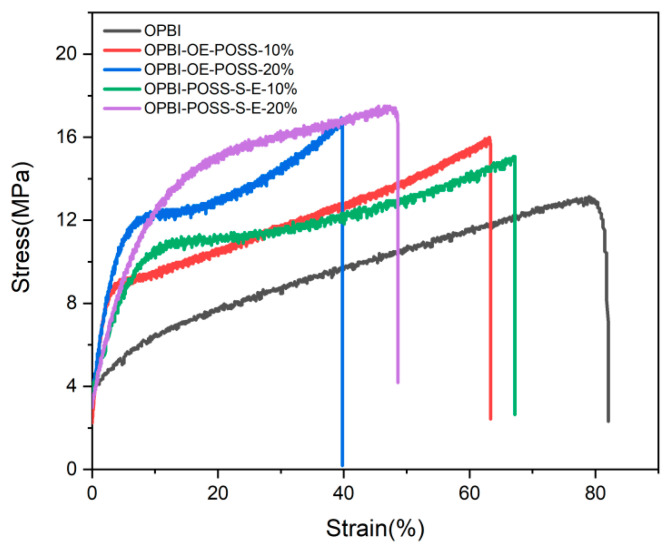
Mechanical properties of different PA-doped membranes at RT.

The operational performance of HT-PEMFCs is significantly influenced by the membrane’s tensile strength. Figure 12 and Table 2 show the mechanical properties of different PA-doped membranes. The tensile strength of crosslinked membranes with POSS is higher than that of OPBI membranes, and the elongation at break is lower than that of OPBI membranes. The tensile strength increases with the increase in POSS, while the elongation at break decreases with the increase in POSS. This is because the dense crosslinked network structure limits the movement of molecular chains and reduces the plasticization of PA. The tensile strength of OPBI-OE-POSS-10% membrane and OPBI-POSS-S-E-10% membrane was 15.9 MPa and 15.1 MPa, respectively, and the tensile strength of OPBI-POSS-S-E-20% membrane was the best, reaching 17.5 MPa. It is higher than the 16.8 MPa of OPBI-OE-POSS-20% membrane because the phosphoric acid doping of OPBI-POSS-S-E-20% membrane is lower than that of OPBI-OE-POSS-20% membrane. In short, the mechanical properties of the series of crosslinked membranes are excellent, which is sufficient for application in HT-PEMFC.

### 3.9. Proton Conductivity

**Figure 13:**
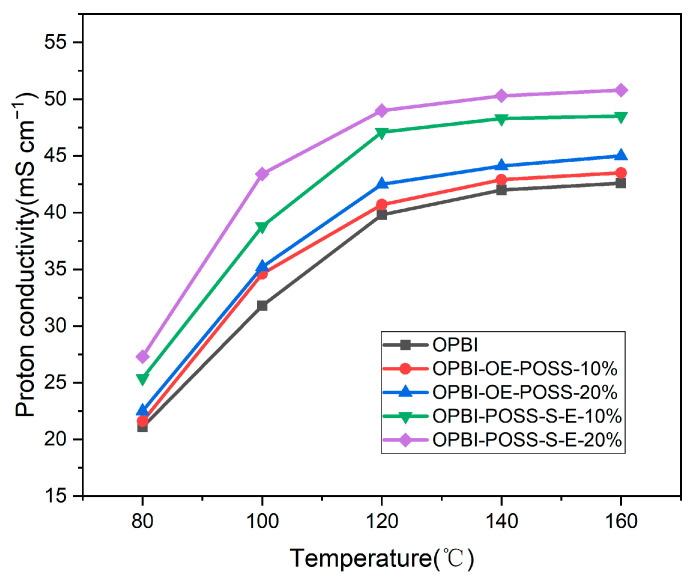
Proton conductivity of PA-doped membranes at different temperature.

Figure 13 shows the proton conductivity of PA-doped membranes at different temperatures. The crosslinked membranes exhibited higher proton conductivity than the OPBI membrane, likely due to the increased PA doping level per unit volume. The proton conductivity of OPBI-POSS-S-E-10% and OPBI-POSS-S-E-20% membranes is higher, reaching 48.5 mS cm^−1^ and 50.8 mS cm^−1^ at 160 °C, respectively. This enhanced conductivity may be attributed to the lower number of epoxy groups on a single POSS-S-E molecule compared to OE-POSS, which reduces the likelihood of self-polymerization reactions during membrane formation. We speculate that this will lead to a reduction in the aggregation of POSS. Therefore, POSS-S-E doping in the membrane is more uniform, which is conducive to the construction of more proton transfer channels and improve the proton conductivity of the membrane.

**Figure 14:**
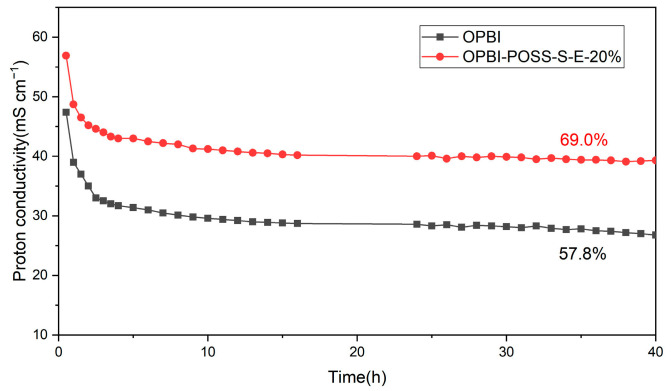
Long-time proton conductivity test of PA-doped membrane at 140 °C.

The proton conductivity stability performance was conducted on PA-doped OPBI membrane and OPBI-POSS-S-E-20% membrane at 140 °C under anhydrous conditions. The results are shown in Figure 14. At the beginning, the proton conductivity dropped sharply because the water in the air absorbed by the membrane quickly evaporated at high temperature. After 40 h, the OPBI-POSS-S-E-20% membrane maintained a proton conductivity of 39.3 mS cm^−1^, corresponding to a retention rate of 69.0%, while the OPBI membrane exhibited a retention rate of only 57.8%. This suggests that the OPBI-POSS-S-E-20% membrane possesses superior acid retention capabilities.

### 3.10. Fuel Cell Performance

**Figure 15:**
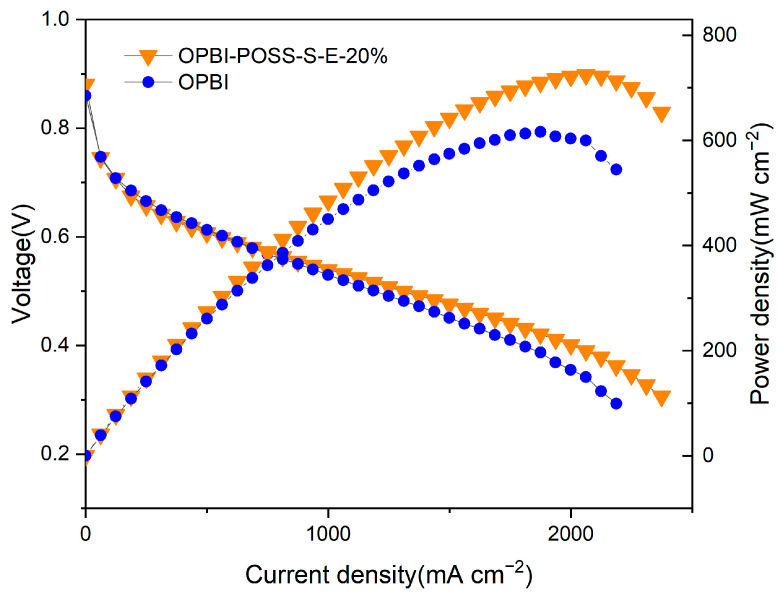
Single cell performance of OPBI-POSS-S-E-20% and OPBI membrane doped with PA at 160 °C.

The OPBI-POSS-S-E-20% membrane with excellent tensile strength and high proton conductivity was assembled by membrane electrolytes. Figure 15 shows the performance of the fuel cell assembled with PA-doped OPBI-POSS-S-E-20% membrane. The thickness of the membrane is 38 μm and the doping amount of PA is 198%, while the OPBI membrane as a control is 41 μm and the doping amount of PA is 235%. The open circuit voltage of OPBI-POSS-S-E-20% membrane and OPBI membrane at 160 °C is 0.88 V and 0.86 V, respectively. The higher open circuit voltage indicates that the membrane has good air tightness and can well separate the cathode and anode of the fuel cell, prevent the direct contact of the fuel gas, and ensure the stability of the fuel cell. At 160 °C, the peak power density of PA-doped OPBI-POSS-S-E-20% membrane reached 724 mW cm^−2^, which was better than that of OPBI membrane of 616 mW cm^−2^ because OPBI-POSS-S-E-20% membrane showed better battery performance due to its higher proton conductivity. It exceeds the performance of advanced phosphoric-acid-doped polybenzimidazole (PBI)-based membranes reported in the recent literature [61,62]. This study mainly focuses on optimizing the content of POSS and its impact on performance and regards the durability during long-term continuous operation as the key direction for future research to verify its commercial feasibility. Additionally, a more systematic study on the relationship between the aggregation structure of POSS and its performance is needed.

## 4. Conclusions

In this work, POSS-SH was successfully synthesized, and OE-POSS and POSS-S-E were subsequently prepared through a thiol-ene reaction between the -SH groups of POSS-SH and the vinyl groups of allyl-glycidyl ether (AGE). These POSS materials were then incorporated into OPBI, resulting in a series of POSS-crosslinked OPBI membranes formed via the reaction between epoxide and imidazole groups during membrane fabrication. Compared to PA-doped OPBI membranes, these crosslinked membranes exhibited improved dimensional stability, mechanical strength, and proton conductivity. The OPBI-POSS-S-E series membranes demonstrated superior oxidation stability compared to the OPBI-OE-POSS series membranes, attributed to the ability of the sulfhydryl groups on POSS to scavenge free radicals. After immersion in Fenton’s reagent at 80 °C for 108 h, the OPBI-POSS-S-E-20% membrane retained 79.4% of its original mass. Furthermore, the proton conductivity of the PA-doped OPBI-OE-POSS-20% membrane reached 50.8 mS cm^−1^ at 160 °C under anhydrous conditions. The peak power density of HT-PEMFC assembled with the OPBI-POSS-S-E-20% membrane reached 724 mW cm^−2^ at 160 °C.

## Figures and Tables

**Table 1:** Acid doping content (ADC), swelling ratio, and *V*_doping_ of different membranes.

Membrane	ADC %	Swelling in Area %	Swelling in Volume %	*V*_doping_ (g cm^−3^)
OPBI	242.0	19.6	96.3	1.05
OPBI-OE-POSS-10%	217.2	13.5	76.1	1.13
OPBI-OE-POSS-20%	200.1	11.2	73.5	1.20
OPBI-POSS-S-E-10%	209.7	12.9	78.2	1.16
OPBI-POSS-S-E-20%	191.8	11.0	68.6	1.22

**Table 2:** Mechanical properties of different PA doped membranes at RT.

Membrane	Tensile Stress/MPa	Elongation/%
OPBI	13.1	82.0
OPBI-OE-POSS-10%	15.9	63.3
OPBI-OE-POSS-20%	16.8	39.8
OPBI-POSS-S-E-10%	15.1	67.2
OPBI-POSS-S-E-20%	17.5	48.6

## Data Availability

The authors confirm that the data supporting the findings of this study are available within the article.

## Supplementary Materials

The following supporting information can be downloaded at: https://www.mdpi.com/article/10.3390/nano16030164/s1, Figure S1. Synthesis process of OPBI. Figure S2. ^1^H NMR of OPBI. Figure S3. ^1^H NMR of POSS-SH, POSS-S-E and OE-POSS.
