# Supplementary material for: Nanostructured POSS Crosslinked Polybenzimidazole with Free Radical Scavenging Function for High-Temperature Proton Exchange Membranes
Source: Nanomaterials (Basel). 2026 Jan 26;16(3):164. doi: 10.3390/nano16030164 (PMC12899568; doi:10.3390/nano16030164)
Supplement: Supplementary file 1 [file nanomaterials-16-00164-s001.zip › nanomaterials-4062300-supplementary.pdf]

Supporting Information

Nanostructured POSS Crosslinked Polybenzimidazole with Free Radical Scavenging Function for High-Temperature Proton Exchange Membranes

Chao Meng [1,2,†], Xiaofeng Hao [1,3,†], Shuanjin Wang [1], Dongmei Han [4], Sheng Huang [1], Jin Li [2], Min Xiao [1,*] and Yuezhong Meng [1,3,4,*]

[1] *The Key Laboratory of Low-Carbon Chemistry & Energy Conservation of Guangdong Province/State Key Laboratory of Optoelectronic Materials and Technologies, School of Materials Science and Engineering, Sun Yat-Sen University, Guangzhou 510275, China*

[2] *GAC AION New Energy Automobile Co., Ltd./Guangzhou Automobile Group Co., Ltd., Guangzhou 511424, China*

[3] *Institute of Chemistry, Henan Provincial Academy of Sciences, Zhengzhou 450014, China*

[4] *School of Chemical Engineering and Technology, Sun Yat-Sen University, Zhuhai 519000, China*

* *Correspondence: stsxm@mail.sysu.edu.cn (M.X.); mengyzh@mail.sysu.edu.cn (Y.M.)*

† *These authors contributed equally to this work.*

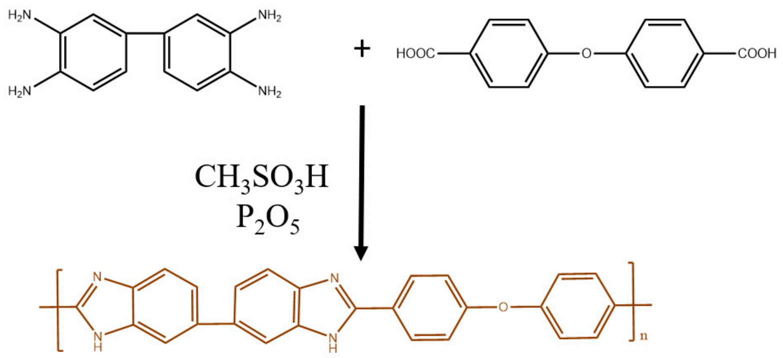

OPBI

Figure S1. Synthesis process of OPBI

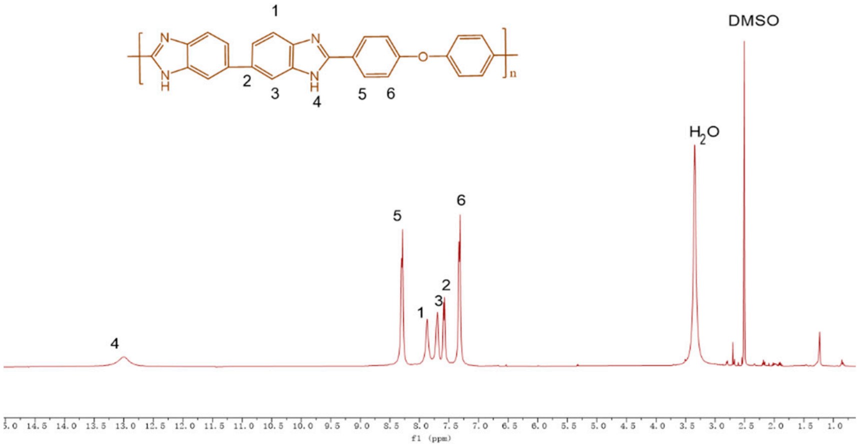

Figure S2. ¹H NMR of OPBI

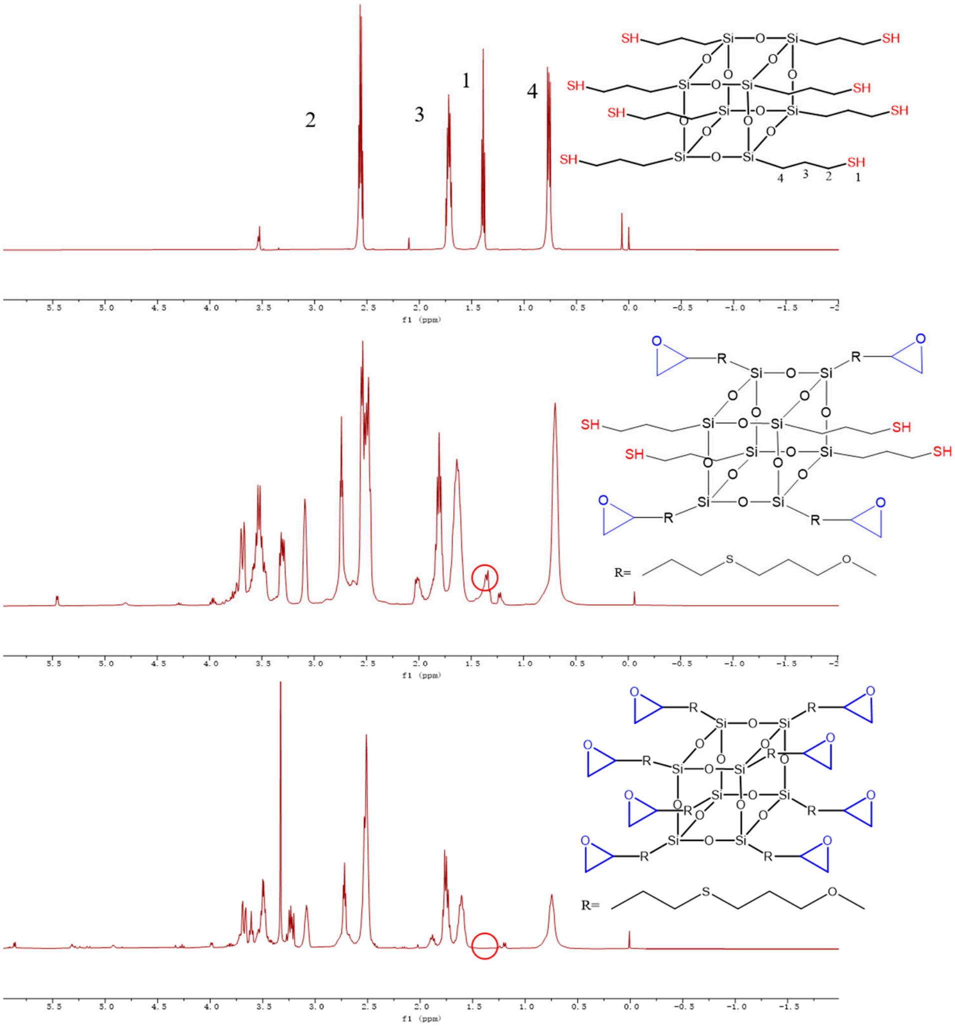

Figure S3. ¹H NMR of POSS-SH, POSS-S-E and OE-POSS
